# Liver-Specific *Bmal1* Depletion Reverses the Beneficial Effects of Nobiletin on Liver Cholesterol Homeostasis in Mice Fed with High-Fat Diet

**DOI:** 10.3390/nu15112547

**Published:** 2023-05-30

**Authors:** Zhitian Lu, Xudong Li, Min Wang, Xiaojun Zhang, Runxuan Zhuang, Fan Wu, Wenxue Li, Wei Zhu, Bo Zhang

**Affiliations:** 1 Food Safety and Health Research Center, School of Public Health, Southern Medical University, Guangzhou 510515, China; lu2t@foxmail.com (Z.L.); lxdnutrition@163.com (X.L.); wangmin274415@163.com (M.W.); zxj201509@163.com (X.Z.); zrx980705@163.com (R.Z.); wf396900@126.com (F.W.); 2 Department of Toxicological and Biochemical Test, Guangzhou Center for Disease Control and Prevention, Guangzhou 510440, China; liwenxue_2000@163.com

**Keywords:** *Bmal1*, nobiletin, cholesterol homeostasis, high-fat diet

## Abstract

Nobiletin (NOB), a naturally occurring small-molecule compound abundant in citrus peels, has displayed potential lipid-lowering and circadian-enhancing properties in preclinical studies. However, the requirement of specific clock genes for the beneficial effects of NOB is not well understood. In the current study, mice with a liver-specific deletion of the core clock component, *Bmal1*—*Bmal1*LKO—were fed a high-fat diet (HFD) ad libitum for eight weeks, while NOB (200 mg/kg) was administered by daily oral gavage from the fifth week and throughout the last four weeks. NOB decreased liver triglyceride (TG) alongside the decreasing mRNA levels of de novo lipogenesis (DNL) genes in both *Bmal1^flox/flox^* and *Bmal1*LKO mice. NOB increased serum very low-density lipoprotein (VLDL) levels in *Bmal1*LKO mice, which was consistent with higher liver *Shp* and lower *Mttp* mRNA expression levels, the key genes that facilitate VLDL assembly and secretion. NOB decreased liver and serum cholesterol levels in the *Bmal1^flox/flox^* mice, consistent with lower *Hmgcr* and higher *Cyp7a1*, *Cyp8b1*, *Gata4* and *Abcg5* mRNA levels in the liver. In contrast, in the *Bmal1*LKO mice, NOB increased *Hmgcr* mRNA levels and had no effect on the above-mentioned genes related to bile acid synthesis and cholesterol excretion, which might contribute to the elevation of liver and serum cholesterol levels in NOB-treated *Bmal1*LKO mice. NOB inhibited hepatic DNL and decreased liver TG levels in HFD-fed mice independently of liver *Bmal1*, whereas liver-specific *Bmal1* depletion reversed the beneficial effects of NOB on liver cholesterol homeostasis. The complex interactions between NOB, the circadian clock and lipid metabolism in the liver warrant further research.

## 1. Introduction

The liver plays a key role in the maintenance of lipid homeostasis. In the post-absorptive state, a large fraction of blood glucose is immediately taken up by the hepatocytes and converted into glycogen [1,2]. After glycogen saturation, any additional glucose enters the metabolic pathways of de novo lipogenesis (DNL) and is esterified into triglycerides (TG) to be exported from the liver, as very low-density lipoprotein (VLDL), to adipose tissues for energy storage [3]. Under fasting conditions, liver glycolysis releases glucose into circulation to maintain glucose homeostasis. After longer fasting periods, hepatocytes take up blood free fatty acids mainly from the lipolysis of adipose tissue, and fatty acid oxidation in the liver is elevated. If the fasting time is longer than >24 h, hepatocytes use intermediate metabolites of fatty acid oxidation to produce ketone bodies [4]. Incompatible anabolism and catabolism in the liver during feeding and fasting states are temporally separated to prevent futile cycles; thus, blood glucose and lipid concentrations are maintained within a narrow range. Temporal variation within a day beyond this range increases the risk of metabolic diseases [5].

Hepatic metabolism is governed by the circadian clock [6]. In mammals, the circadian clock consists of interlocked transcription–translation feedback loops that exhibit auto-regulatory oscillations within a 24-h period. The core components of the circadian clock (i.e., the so called “clock genes”) include a series of transcription factors and nuclear receptors. The transcription factor called the brain and muscle aryl hydrocarbon receptor nuclear translocator-like protein 1 (BMAL1) and circadian locomotor output cycles kaput (CLOCK) heterodimers (BMAL1:CLOCK) bind to enhancer boxes (E-boxes) in the promoters and drive the expression of their target genes, including their own repressors—period circadian protein homologues 1, 2, and 3 (PER1, PER2, and PER3) and cryptochromes 1 and 2 (CRY1 and CRY2), which make up the core negative-feedback loop. Further, BMAL1:CLOCK also activates the expression of retinoic acid receptor-related orphan receptors (RORα, RORβ and RORγ) and REV-ERB (REV-ERBα and REV-ERBβ), which antagonistically activate or repress *Bmal1* expression by competitively binding to ROR/REV-ERB-response elements and then form the secondary stabilization feedback loop. More loops are yet to be discovered [7]. Together, these interlocked feedback loops can drive the expression of a large number of target genes called “clock-controlled genes”, which involves many metabolic regulators such as peroxisome proliferator-activated receptors (PPARs) [8], sterol regulatory element binding protein (SREBP) [9], and AMP-activated protein kinase (AMPK) [10,11], thereby coupling the circadian clock and nutrient and energy metabolism. For example, Pan et al. [12] showed that the circadian clock regulates plasma lipids, and the deregulation of the circadian rhythms causes hyperlipidemia in mice. They also found that BMAL1 orchestrates different steps in the biosynthesis of ApoB-containing lipoproteins by modulating the expression of a small heterodimer partner (*Shp*) and cAMP-responsive element-binding protein H [13]. However, the relationship between circadian rhythms and metabolism in specific tissues, such as the liver, is not well understood.

Mice with liver-specific deletions of clock genes have been shown to develop hyperlipidemia, suggesting a strong mechanistic link between proper clock function and metabolism. Given that BMAL1 is viewed as an indispensable transcriptional activator of the clock system, liver-specific *Bmal1* knockout (*Bmal1*LKO) mice have higher TG, cholesterol [13,14,15,16,17], and free fatty acid [17] in plasma and elevated TG levels in the liver [15]. Daily temporal variances in TG [13,15,16,17] and high-density lipoprotein (HDL) [16] were more apparent in *Bmal1*LKO mice than in wild-type (WT) controls, while the temporal differences in total cholesterol [13,15,16,17] and low-density lipoprotein (LDL) [16] exhibited less change. Additionally, *Bmal1*LKO mice fed ad libitum also had elevated insulin levels and did not show glucose intolerance but exhibited exaggerated glucose clearance and hypoglycemia which was restricted to the fasting phase [14,16,18].

Compelling evidence suggests that disruption of the circadian clock is the cause of metabolic diseases [19,20]. However, these studies raise the possibility of a novel therapeutic strategy for metabolic disorders: the development of small molecules to manipulate the circadian clock—for example, the REV-ERB agonist SR9009, GSK4112 and the RORα inverse agonist SR3335 were used to improve metabolic disorders in rodents [21,22]. Nobiletin (NOB), a natural polymethoxyflavonoid abundant in citrus peel, has lipid-lowering, insulin-sensitizing, and antidiabetic effects in mice and in vitro cell lines [23,24,25]. Dried tangerine peel has been used in cooking, teas, and traditional drugs in East-Asian regions for hundreds of years. He et al. [26] revealed that NOB-activated RORs stabilize the core BMAL1:CLOCK transcriptional feedback loop, enhance the circadian clock system, and protect against metabolic disorders in a clock gene-dependent manner in both diet-induced obese (DIO) and *db*/*db* mice. Solt et al. [27] found that the treatment of high-fat DIO mice with a small-molecule agonist of REV-ERBα (SR9009) for 12 days decreased obesity by reducing fat mass and remarkably improving dyslipidemia and hyperglycemia. Given the powerful compensatory mechanism of the circadian rhythm, it is not surprising that the agonists of both an activator (RORs) [26] and repressor (REV-ERBα) [27] showed similar beneficial metabolic effects, but the process raises a concern regarding the off-target effects of these small molecules.

In the present study, we used *Bmal1*LKO mice to observe the effects of NOB on liver lipid homeostasis under a high-fat diet (HFD). We found that NOB inhibited hepatic DNL and decreased liver TG levels in HFD-fed mice independent of liver *Bmal1*, whereas liver-specific *Bmal1* depletion reversed the beneficial effects of NOB on liver cholesterol homeostasis.

## 2. Materials and Methods

### 2.1. Chemicals

Nobiletin (CAS: 478-01-3; purity: ≥98%) was purchased from Sichuan Vikki Biotechnology Co. Ltd. (Sichuan, China). The NOB solution was prepared by dissolving 200 mg of NOB in 10 mL of 0.5% sodium carboxymethyl cellulose, and the reference group received 0.5% sodium carboxymethyl cellulose.

### 2.2. Animal

WT specific pathogen-free (SPF) C57BL/6J male mice at 6–7 weeks of age were purchased from Guangdong Sja Biotechnology Co., Ltd. (Guangzhou, China). Furthermore, *Bmal1^flox/flox^* mice with a C57BL/6J genetic background were purchased from Cyagen Biosciences, Inc. (Suzhou, China). The *Bmal1*LKO mice were obtained by mating the *Bmal1^flox/flox^* mice with AlbuminCre mice. At four weeks after birth, the ear tissue of the mice was collected and directly lysed by 100 μL lysis buffer from the mouse direct polymerase chain reaction (PCR) kit (Bimake). The tissue lysate was used as a PCR template to amplify the editing site, followed by gel electrophoresis of the products (Figure S1). The primers used are listed in Supplementary Materials Table S1. The success of liver-specific *Bmal1* knockout was determined by western blot (WB) analysis of BMAL1 expression levels (Figure S1) for each organ and primary hepatocyte. Detailed information on the method of primary hepatocyte isolation and WB is in the Supplementary Materials. All mice with similar genotype were housed (five mice per cage) in a SPF animal facility and fed ad libitum under a 12 h:12 h light–dark cycle, at a temperature of 22 ± 2 °C and relative humidity of 30–60%. The protocols for animal experiments were approved by the Laboratory Animal Center of South China Agricultural University (protocol number: 2021D143).

### 2.3. Rhythm Detection Program

After two weeks of acclimatization, 35 WT and 35 *Bmal1*LKO male mice fed with normal chow (NC) were euthanized every 4 h for a total of 24 h, namely ZT0, ZT4, ZT8, ZT12, ZT16, ZT20, and ZT24 (the time of ‘lights on’ usually defines zeitgeber time zero ‘ZT0’, and the time of ‘lights off’ defines ZT12). All mice were fasted for 24 h before sacrifice.

### 2.4. NOB Treatment Experiment

Ten male *Bmal1^flox/flox^* and 10 male *Bmal1*LKO mice were fed with HFD (D12492, Supplementary Materials Table S2) for eight weeks. Mice with each genotype were randomized into two groups. During the last four weeks (i.e., from the fifth week of HFD), the mice were treated with either reference or NOB (200 mg/kg/day) via oral gavage every day in the daily time window of ZT4–ZT5 until the last day of HFD. The dose was chosen because previous in vivo studies using similar overall amounts of NOB (100–400 mg/kg/day) had found their beneficial effects on liver lipid metabolism [26,28]. All the mice were fasted for 12 h before sacrifice.

### 2.5. Serum and Liver Collection

Blood was collected and the liver was immediately removed after anesthetization with 1% sodium pentobarbital. The weight of the liver was recorded. Part of the right lobe of the liver was fixed with 4% paraformaldehyde, and the remaining part of the liver was snap-frozen in liquid nitrogen and stored at −80 °C until analysis. The blood was centrifuged at 3000 rpm for 15 min at room temperature, and the supernatant was separated to obtain serum, which was snap-frozen in liquid nitrogen and stored at −80 °C until analysis.

### 2.6. Assay of Biochemical Parameters

Serum TG, TC, LDL, HDL, VLDL, glucose (Glu), free fatty acids (FFA), alanine aminotransferase (ALT), aspartate aminotransferase (AST) and Total bile acid (TBA) levels were determined using commercial kits (Nanjing Jiancheng Bioengineering Institute, Nanjing, China). The supernatant was obtained by homogenizing 50 mg of liver tissue using 1 mL of lysis solution, water bathing at 70 °C for 10 min, and centrifuged at 2000 rpm for 5 min at room temperature. Liver TG, TC, TBA and FFA levels in the supernatant were determined using commercial kits (Beijing Applygen Technologies Inc. Beijing, China), and the bicinchoninic acid protein quantification method (Beyotime Institute of Biotechnology, Shanghai, China) was used to quantify hepatic tissue protein in the supernatant to calculate the liver TG and TC concentrations.

### 2.7. Liver Histology

After fixation, being embedded in paraffin and sectioned at 5 µm, liver morphology and TG content were assessed using hematoxylin and eosin (H&E) and Oil red O of staining tissue sections, respectively. Images were captured using an Axio Examiner light microscope (Carl Zeiss AG, Oberkochen, Germany). Quantification of hepatic TG content in Oil red O-stained samples was performed using the ImageJ morphometric software (National Institutes of Health, Bethesda, MD, USA).

### 2.8. Quantitative Reverse Transcription-Polymerase Chain Reaction (qRT-PCR)

Total RNA was extracted from frozen liver tissues using TRIzol (Invitrogen), and cDNA was synthesized by reverse transcription. The mRNA expression levels of circadian rhythm- and metabolism-related genes were assessed using gene-specific primers and RT-PCR with a SYBR Green Master PCR kit (Accurate Biotechnology [Hunan] Co., Ltd. Changsha, China) on ABI QuantStudio 6 Flex RT-PCR Systems (Thermo Fisher Scientific, Waltham, MA, USA). Gene expression levels were normalized by *β-actin* (*Actb*) expression. The primer sequences used are listed in Supplementary Materials Table S2. The PCR reaction conditions were pre-denaturation at 95 °C for 300 s, denaturation at 95 °C for 20 s, annealing at 60 °C for 20 s, and extension at 72 °C for 20 s and 40 cycles. After amplification, the temperature was lowered to 60 °C and increased to 95 °C to denature the DNA product. The relative quantification method (2^−ΔΔCt^) was used to compare differences in gene expression levels between the groups.

### 2.9. Statistical Analyses

The results were statistically analyzed using SPSS software (version 20.0). A 24-h rhythmicity was detected using Sigma Plot 14.0 (Systat Software, Inc. San Jose, CA, USA). Student’s *t*-test was used to compare the two groups. The Fisher’s least significant difference was used for analysis of variance (ANOVA) post-hoc test. A two-way ANOVA was used to analyze the interaction between genotype and NOB treatment. Statistical significance was considered at *p* < 0.05.

## 3. Results

### 3.1. Liver-Specific Bmal1 Knockout Remodels 24-h Rhythmicity of Liver and Serum Metabolic Parameters in NC-Fed Mice

**Figure 1:**
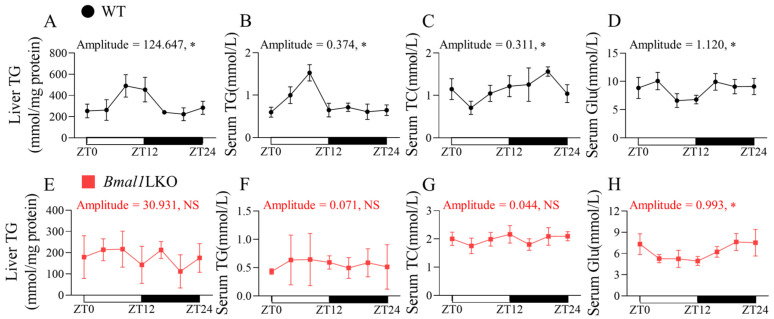
Effects of liver-specific *Bmal1* knockout on lipid and glucose oscillation in WT and *Bmal1*LKO mice: liver TG (**A**,**E**), serum TG (**B**,**F**), serum TC (**C**,**G**) and serum Glu (**D**,**H**) levels after 24-h fasting in NC-fed WT and *Bmal1*LKO mice at seven time points. Data are presented as mean ± SD (*n* = 5 per group at each time point). * *p* < 0.05 indicates that the 24-h rhythmicity was significant.

To determine whether the liver weight and serum lipids exhibited 24-h oscillations after *Bmal1* deletion in the liver, we sacrificed the WT and *Bmal1*LKO mice fed with NC at seven time points in a 24-h period. There was no significant rhythmicity in liver weight in either the WT or *Bmal1*LKO mice (Figure S2B,E). Under NC feeding, liver TG, serum TG, serum TC, and serum Glu levels in the WT mice showed a 24-h rhythmicity (Figure 1). However, in the *Bmal1*LKO mice, only serum Glu showed a 24-h oscillation (Figure 1H).

### 3.2. Liver-Specific Bmal1 Knockout Altered the Expression of Clock Genes and Clock-Controlled Genes in the Liver

To explore the underlying mechanism by which hepatic *Bmal1* deletion leads to the loss of rhythmicity of liver and blood lipids, we used qRT-PCR to detect the expression levels of clock and clock-controlled genes in the liver over a 24-h period.

**Figure 2:**
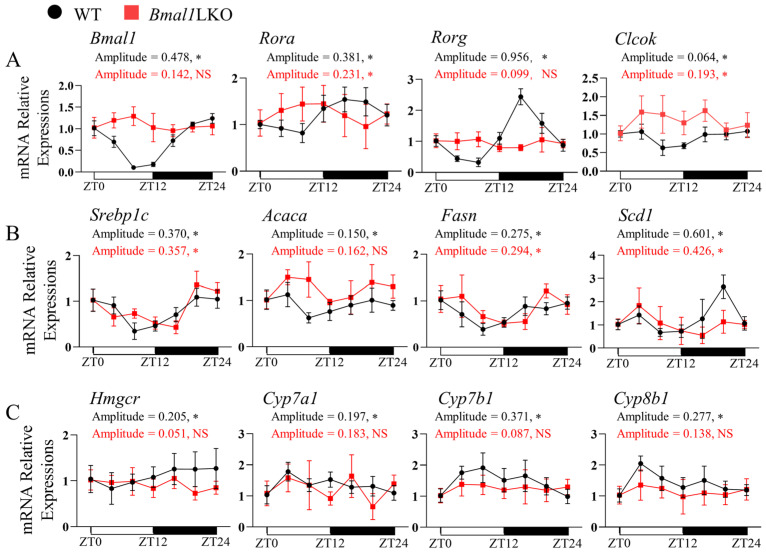
Effects of liver-specific *Bmal1* knockout on the expression of clock and clock-controlled genes in the liver of mice: qRT-PCR analyses of the mRNA expression of clock genes (**A**), DNL genes (**B**), and cholesterol metabolism genes (**C**) in the livers of NC-fed WT and *Bmal1*LKO mice at seven time points after a 24-h fasting. Data are presented as mean ± SD (*n* = 5 per group at each time point). * *p* < 0.05 indicates that the 24-h rhythmicity was significant.

The core clock, DNL, and cholesterol metabolic genes in the WT mice showed a significant 24-h oscillation in mRNA levels; however, in *Bmal1*LKO mice, the expression levels of core clock genes (*Bmal1*, *Clock*, *Cry1*, *Cry2*, and *Rorg*) lost rhythmicity, and the amplitudes of *Rev-erba*, *Per1* and *Per2* decreased (Figure 2A and Figure S3A). We also examined the expression of hepatic clock-controlled genes related to lipid metabolism. The mRNA rhythmicity of *Ppara*, *Pparg*, *Ppargc1a*, *Hmgcr*, *Cyp7a1*, *Cyp7b1*, *Cyp8b1* disappeared, whereas the oscillation of DNL genes (*Srebp1c*, *Acaca*, *Fasn*, and *Scd1*) was unchanged (Figure 2B,C and Figure S3B). These results indicated that in mice with hepatic *Bmal1* deficiency, the oscillations of liver core clock genes were weakened, and the expression of cholesterol metabolic genes lost rhythmicity, while the daily expression profiles were almost unchanged.

### 3.3. NOB Prevents HFD-Fed Bmal1^flox/flox^ and Bmal1LKO Mice from Gaining Body Weight and White Adipose Tissue Content

**Figure 3:**
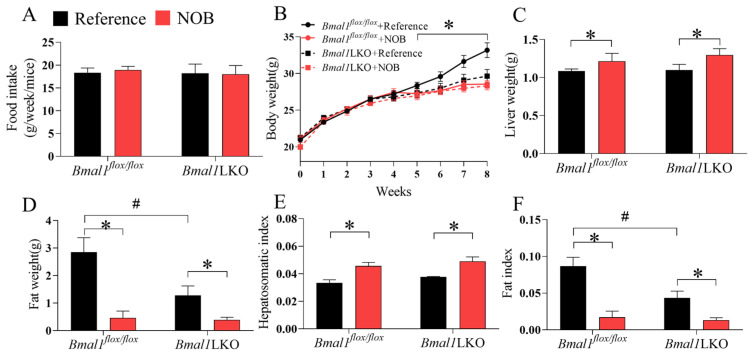
Effects of Nobiletin (NOB) on energy intake, body weight gain, liver weight and fat weight in HFD-fed mice: energy intake (**A**), body weight (**B**), liver weight (**C**), fat weight (**D**), hepatosomatic index (**E**), and fat index (**F**) in HFD-fed mice with *Bmal1^flox/flox^* (*flox/flox*) and *Bmal1*LKO (LKO) mice with reference or NOB treatment. Data are presented as mean ± SD (*n* = 5 per group). * *p* < 0.05, *flox/flox*.Reference versus *flox/flox*.NOB or LKO.Reference versus LKO.NOB. # *p* < 0.05, *flox/flox*.Reference versus LKO.Reference.

We investigated the role of the circadian clock in the protective effects of NOB on lipid metabolism. An essential component of the circadian rhythm, *Bmal1*, was specifically knocked out in the liver to generate liver-specific *Bmal1* deficiency mice (*Bmal1*LKO). The *Bmal1^flox/flox^* mice served as controls. Mice of both genotypes were treated with either reference or NOB via oral gavage every day for four weeks from weeks 5 to 8, under a total of eight weeks of HFD. There was no significant difference in food intake between the *Bmal1^flox/flox^* and *Bmal1*LKO mice (Figure 3A). Compared to the *Bmal1^flox/flox^* mice, the body weight gain of the *Bmal1*LKO mice began to slow from week 4. NOB significantly decreased the body weight gain in the *Bmal1^flox/flox^* mice (Figure 3B); it increased liver weight (Figure 3C), but significantly decreased white adipose tissue (Figure 3F) in both the *Bmal1^flox/flox^* and *Bmal1*LKO mice.

### 3.4. Effects of NOB on Liver Pathological Features in HFD-Fed Mice

**Figure 4:**
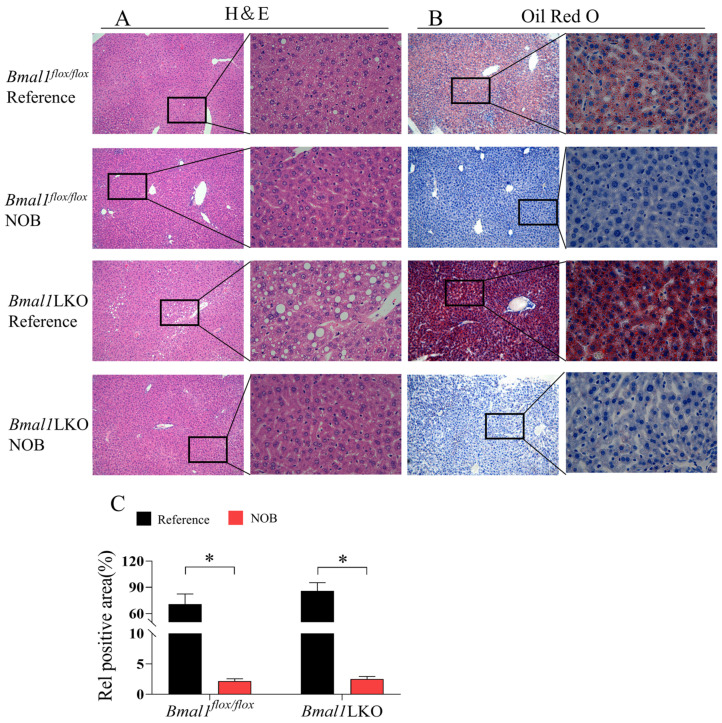
NOB decreased NAFLD activity score and hepatic lipid accumulation in HFD-fed mice: representative images of H&E staining (**A**), Oil Red O staining (**B**) and oil red O staining quantitative analysis (**C**) of the livers of HFD-fed *Bmal1^flox/flox^* and *Bmal1*LKO mice after eight weeks of treatment. Data are presented as mean ± SD (*n* = 5 per group). (**A**): 10× objective, 100× total magnification (**left**) and 40× objective, 400× total magnification (**right**), (**B**): 10× objective, 100× total magnification (**left**) and 40× objective, 400× total magnification (**right**). * *p* < 0.05, *flox/flox*.Reference versus *flox/flox*.NOB or LKO.Reference versus LKO.NOB.

Hematoxylin and eosin staining showed that the nuclei of hepatocytes in the *Bmal1^flox/flox^* mice were large, round, and centered, with clearly visible nucleoli and a few fat vacuoles. In the *Bmal1*LKO mice, the hepatocytes nuclei were offset from the cell center by mixed fat vacuolation and fat deposits. NOB significantly restored the hepatocyte morphology and reduced the number of fat vacuoles in both the *Bmal1^flox/flox^* and *Bmal1*LKO mice (Figure 4A). Compared to the *Bmal1^flox/flox^* mice, the red-stained liver area of the *Bmal1*LKO mice increased significantly, and the quantitative results of Oil red O staining showed that liver TG content in the *Bmal1*LKO mice was significantly higher than that in the *Bmal1^flox/flox^* mice, whereas NOB significantly decreased hepatic TG in the *Bmal1^flox/flox^* and *Bmal1*LKO mice (Figure 4B,C).

### 3.5. NOB Improves Liver and Serum Lipid Homeostasis in HFD-Fed Mice in a Hepatic Bmal1-Dependent or Bmal1-Independent Manner

**Figure 5:**
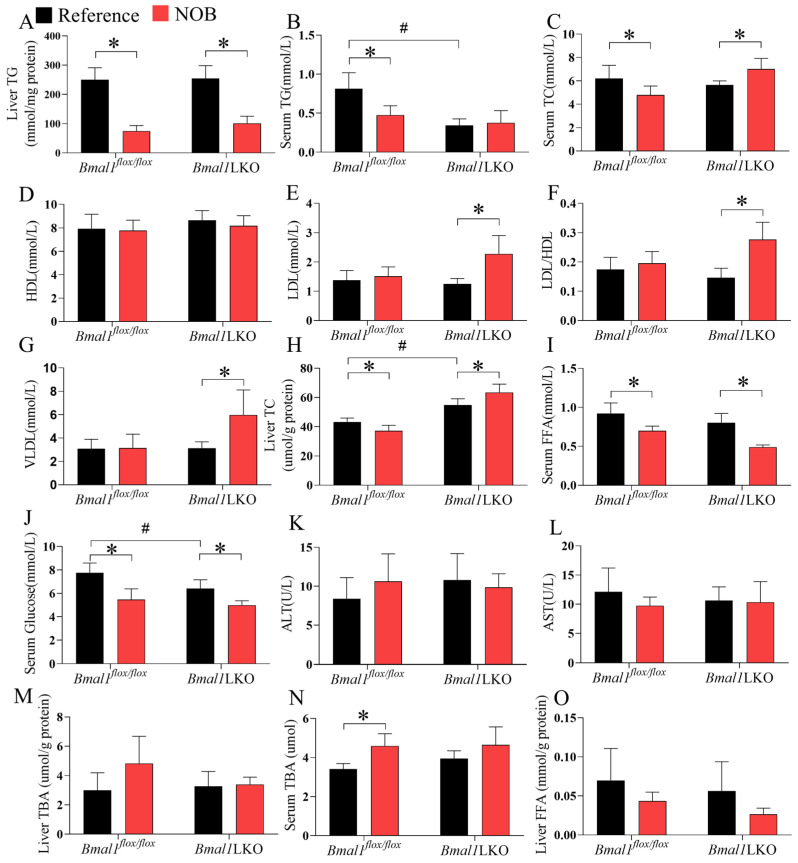
NOB improves liver and serum lipid and glucose homeostasis in HFD-fed mice in a hepatic *Bmal1*-dependent or *Bmal1*-independent manner: liver TG (**A**), serum TG (**B**), serum TC (**C**), serum HDL (**D**), serum LDL (**E**), serum LDL/HDL ratio (**F**), serum VLDL (**G**), liver TC (**H**), serum FFA (**I**), serum glucose (**J**), serum ALT (**K**), serum AST (**L**)*,* liver TBA (**M**), serum TBA (**N**) and liver FFA (**O**) levels after 12-h fasting in HFD-fed *Bmal1^flox/flox^* and *Bmal1*LKO mice with reference or NOB treatment. Data are presented as mean ± SD (*n* = 5 per group). * *p* < 0.05, *flox/flox*.Reference versus *flox/flox*.NOB or LKO.Reference versus LKO.NOB. # *p* < 0.05, *flox/flox*.Reference versus LKO.Reference.

NOB significantly reduced liver TG levels in the *Bmal1^flox/flox^* and *Bmal1*LKO mice, but only reduced serum TG and TC levels in the *Bmal1^flox/flox^* mice (Figure 5A–C). However, in the *Bmal1*LKO mice, NOB increased serum TC, LDL, and VLDL levels (Figure 5D–G). Similar to serum TC, NOB decreased liver TC levels in the *Bmal1^flox/flox^* mice, but increased them in the *Bmal1*LKO mice (*p* for interaction between genotype and NOB treatment was 0.001, Figure 5H). NOB decreased the serum FFA and glucose levels in the *Bmal1^flox/flox^* and *Bmal1*LKO mice (Figure 5I,J). To avoid the concern of liver injury caused by NOB, we tested serum ALT and AST and found that NOB does not cause liver injury (Figure 5K,L). Additionally, NOB had no effects on liver TBA in either mice (Figure 5M), but increased the serum TBA level in *Bmal1^flox/flox^* mice and had no effects in *Bmal1*LKO mice (Figure 5N). NOB did not decrease hepatic FFA levels significantly in either *Bmal1^flox/flox^* or *Bmal1*LKO mice, although there was a decreasing trend in NOB-treated mice (Figure 5O). These results suggest that although liver *Bmal1* deletion aggravates hepatic lipid accumulation in HFD-fed mice, NOB reduced hepatic TG accumulation independently of *Bmal1*, but the beneficial effects on liver cholesterol metabolism depend on *Bmal1*.

### 3.6. NOB Altered Liver Expression of Clock Genes and Clock-Controlled Genes in HFD-Fed Bmal1^flox/flox^ and Bmal1LKO Mice

**Figure 6:**
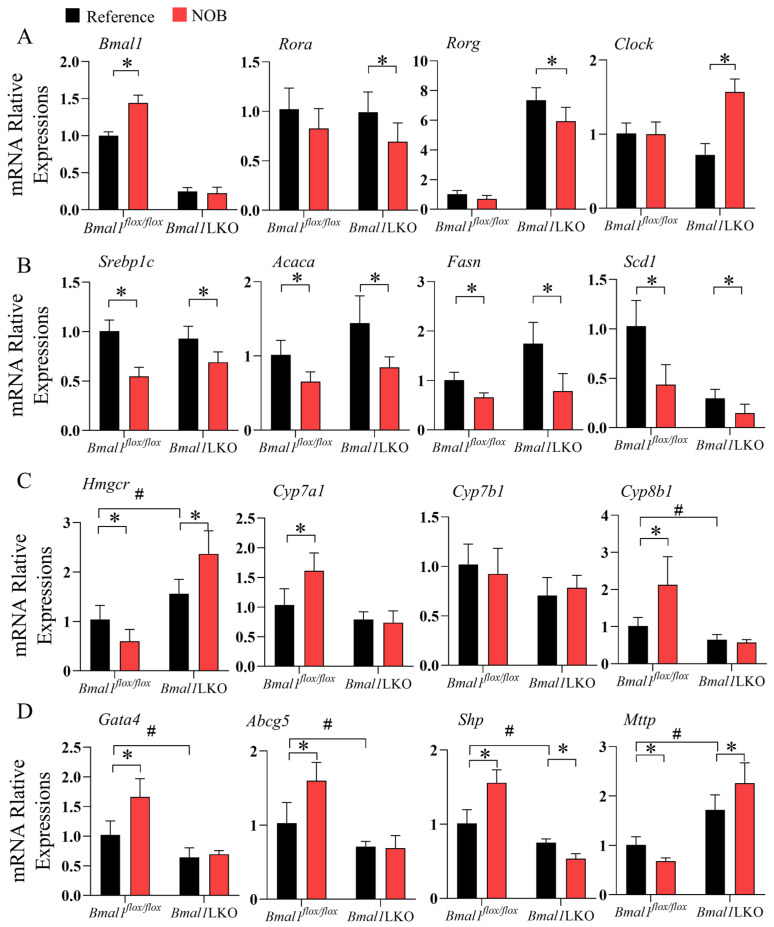
NOB altered the expression of clock and lipid metabolism genes in the liver of HFD-fed *Bmal1^flox/flox^* and *Bmal1*LKO mice: qRT-PCR analyses of mRNA expression of clock genes (**A**), lipid synthesis genes (**B**), and cholesterol metabolic and lipoprotein secretion genes (**C**,**D**) after a 12-h fasting in the liver of HFD-fed *Bmal1^flox/flox^* and *Bmal1*LKO mice after eight weeks of treatment. Data are presented as mean ± SD (*n* = 5 per group). * *p* < 0.05, *flox/flox*.Reference versus *flox/flox*.NOB or LKO.Reference versus LKO.NOB. # *p* < 0.05, *flox/flox*.Reference versus LKO.Reference.

Given that NOB treatment had different effects on body weight, and on liver and serum lipid levels in the *Bmal1^flox/flox^* and *Bmal1*LKO mice, we measured the mRNA expression levels of liver clock and clock-controlled genes involved in lipid metabolism. NOB significantly increased the expression of *Bmal1* in the livers of *Bmal1^flox/flox^* mice, and other clock genes—including *Rev-erba*, *Rora*, and *Rorg*—were significantly downregulated by NOB in the HFD-fed *Bmal1*LKO mice, but not in the *Bmal1^flox/flox^* mice (Figure 6A and Figure S4A).

Next, we examined liver lipid metabolic genes. Consistent with the TG levels in the liver, expression of genes promoting DNL, including *Srebp1c*, *Acaca*, *Fasn*, *Scd1*, *Dgat2*, and *Elovl3*, were significantly downregulated by NOB treatment in both *Bmal1^flox/flox^* and *Bmal1*LKO mice (Figure 6B and Figure S4B); NOB significantly downregulated the expression level of *Pparg* in the *Bmal1^flox/flox^* mice, but upregulated *Pparg* expression in the *Bmal1*LKO mice (Figure S4B). As for hepatic lipid oxidation, NOB did not change the level of the β oxidation rate-limiting enzyme, *Cpt1a*, but significantly downregulated the expression level of *Ppara* in the *Bmal1^flox/flox^* mice and of *Acox1* in the *Bmal1*LKO mice (Figure S4C).

The expression of *Hmgcr* gene, which encodes HMG-CoA reductase, was reduced in the *Bmal1^flox/flox^* mice, but increased in the *Bmal1*LKO mice (Figure 6C). Rate-limiting enzymes in the bile acid synthesis pathway, including *Cyp7a1*, *Cyp7b1*, and *Cyp8b1*, were also measured. NOB significantly increased the expression levels of *Cyp7a1* and *Cyp8b1* in the HFD-fed *Bmal1^flox/flox^* mice (Figure 6C) but not in the *Bmal1*LKO mice, suggesting that the effects of NOB on the conversion of cholesterol to bile acids in the liver were dependent on *Bmal1*. Moreover, NOB significantly increased the expression of *Gata4* and *Abcg5* in the *Bmal1^flox/flox^* mice. Additionally, *Gata4* and *Abcg5* are responsible for the excretion of cholesterol into the gallbladder and intestine, suggesting that NOB promotes hepatic cholesterol export [12]. The expression level of *Mttp*, a key enzyme in VLDL secretion, decreased in the NOB-treated *Bmal1^flox/flox^* mice but increased in the *Bmal1*LKO mice (*p* value for the interaction between genotype and NOB treatment was 0.002). Significantly, *Shp* is a transcriptional repressor of *Mttp* and its expression is opposite to *Mttp*.

## 4. Discussion

To explore whether the protective effects of NOB against HFD-induced lipid metabolic dysregulation were dependent on the liver core clock gene, *Bmal1*, *Bmal1*LKO and control mice were fed with HFD for eight weeks, accompanied by NOB administration in the last four weeks. We observed that the beneficial effects of NOB on liver cholesterol and TG metabolism differed according to the genotype of liver BMAL1, which was consistent with the mRNA expression levels of genes involved in liver lipid metabolic processes, including DNL, VLDL assembly and secretion, cholesterol synthesis, and efflux.

The *Bmal1*LKO mice exhibited lower fasting serum glucose levels than the controls under an eight-week HFD, which is consistent with previous findings that liver-specific deletion of *Bmal1* leads to increased glucose clearance [14,16,18]. The effects of *Bmal1* knockout on liver and serum lipids are inconsistent with previous studies [14,16,29], and might, partially depend on the circadian time of sampling and fasting duration before sampling given that the daily oscillations of lipid metabolism under feeding and fasting states are more complex. Whole-body deletion of *Bmal1* in mice causes behavioral arrhythmias, accompanied by many adverse effects related to early aging [30]. Tissue-specific deletion of *Bmal1* in the liver [17,18,29], pancreas [31], adipocytes [32], skeletal muscle [33,34], and intestines [35] recapitulates the discrete elements of metabolic disorders under various environmental stimuli.

Among the WT mice fed with HFD, four-week NOB treatment significantly attenuated body weight gain and reduced fat mass without altering food intake. NOB also improved lipid homeostasis in the WT mice, thereby showing fewer lipid deposits in the liver and lower liver and serum TC and TG. The protective effects of NOB against HFD-induced metabolic dysregulation in the WT mice are consistent with previous reports [26,28,36]. We also confirmed that the downregulation of genes involved in fatty acid synthesis in the liver by NOB might contribute to the improvement of both hepatic steatosis and dyslipidemia [28,36].

Unexpectedly, NOB also decreased liver lipid deposits and TG content in the *Bmal1*LKO mice, as a result of the tight co-regulation of metabolic and circadian systems [37]. Previous studies by Zhang et al. [38] showed that hepatic BMAL1 promotes DNL via insulin-mTORC2-AKT signaling during refeeding in mice, and that the protective action of BMAL1 against alcoholic liver disease in mice depends on its ability to couple carbohydrate response element binding protein (ChREBP)-induced DNL with PPARα-mediated fatty acid oxidation [29]. Recent evidence has shown that PPARα has a direct positive-regulatory feedback loop with BMAL1 in the liver [8]. In contrast, although NOB potently protects against metabolic syndrome in DIO mice and *db/db* mice in a clock-dependent manner, it directly binds to and activates RORs, which are components within the forward limb of the circadian clock system, and enhances *Bmal1* transcription [26]. Additionally, the essential role of *Bmal1* in circadian mechanisms has been identified, but more factors are yet to be identified. For example, among 18,079 coding transcripts identified by RNA sequencing in WT mice, 2010 were *Bmal1*-dependent oscillating transcripts. Of these, only approximately 10.8% (218) oscillated in liver-RE mice, which were generated with rhythmic re-expression of BMAL1 exclusively in the liver on an arrhythmic *Bmal1*-null mouse background [39], suggesting that additional signals beyond *Bmal1* are essential for circadian regulation in the liver.

Although existing studies illustrate that interventions targeting circadian clocks, such as time- and calorie-restricted feeding, genetic manipulation, and drugs, have promising potential as preventive or therapeutic strategies to combat metabolic diseases [21], the results are not consistent. Mice fed with HFD showed profound circadian remodeling of metabolic markers and transcription networks of lipid metabolic genes in the liver, despite only modest or no changes in the diurnal expression of core clock genes, including *Bmal1*, *Clock*, and *Per2* [40,41]. Similarly, a ketogenic diet drives the liver-specific oscillation of PPARα and its target genes without altering the expression profiles (phase and amplitude) of *Bmal1*, *Cry1*, *Per2*, and *Rev-erba* [42]. Time-restricted feeding (TRF) can drive and remodel the daily rhythms of many pathways responsible for hepatic glucose and lipid metabolism in terms of gene expression and metabolic processes; however, in mice with whole-body or liver-specific deletion of *Bmal1* or other circadian clocks, TRF can reduce the accumulation of hepatic lipids and prevent obesity and metabolic syndrome [14].

In the current study, NOB decreased liver and serum cholesterol levels in HFD-fed WT mice. Liver gene expression levels revealed potential underlying mechanisms related to the inhibition of cholesterol synthesis and VLDL assembly and the induction of bile acid conversion and efflux. These NOB-induced effects are dependent on liver *Bmal1* because null or even opposite effects were observed in the *Bmal1*LKO mice.

Hepatic cholesterol and bile acid metabolism are subject to circadian regulation [9,43,44]; however, the physiological significance of their daily oscillations remain unclear [45]. Pan et al. [12] found that global or liver *Bmal1* deficiency affects two different pathways of liver cholesterol clearance by increasing cholesterol secretion via VLDL through the suppression of *shp* and induction of *Mttp* expression. This reduces cholesterol efflux to bile due to lower expression of *Gata4*, *Abcg5* and *Abcg8*. The effects of *Bmal1* deficiency on gene expression were similar to those of NOB treatment in our study.

Although BMAL1 is considered an indispensable transcriptional activator of the clock system, its functions are not fully understood. There is compelling evidence suggesting that BMAL1 has non-circadian functions (i.e., functions that are not related to its rhythmic expression). In global *Bmal1* knockout mice, constant re-expression of BMAL1 without daily oscillation in the brain and muscle tissues rescues some but not all disrupted rhythmic behaviors [46], suggesting that the rhythmicity of *Bmal1* mRNA expression levels is not essential for rhythmic behavior. Li et al. [47] found that a liver-enriched lncRNA regulator of hyperlipidemia binds directly to heterogeneous nuclear ribonuclear protein U (hnRNPU), and that the hnRNPU can transcriptionally activate *Bmal1*, leading to the inhibition of VLDL secretion in hepatocytes, and thereby proposing a new functional model of BMAL1 in lipid metabolism.

As an integrated or indispensable component of the clock system, the effect of *Bmal1* deficiency may reflect the dysregulation of all circadian regulatory mechanisms. However, compensatory changes in the paralogs of other core clock components may buffer the effects of *Bmal1*. With these strong compensatory mechanisms, it is not surprising that small molecules targeting either the negative limb (e.g., REV-ERB agonist) [27] or the forward limb (e.g., RORs agonist) [26] of the clock system can achieve similar beneficial effects on metabolism in DIO mice.

Many studies have suggested the possibility of using the circadian clock as a therapeutic target, and developing drugs to manipulate the biological clock for the treatment of metabolic diseases [21]. Our study shows the complex interactions between NOB, *Bmal1*, and lipid metabolism in the liver. Therefore, the beneficial and harmful effects of NOB as an enhancer of circadian rhythms should be fully studied.

A drawback of our studies is that all observations were performed at only one time point (ZT4–ZT5) during the light period, which might not be the optimal time to find differences for all lipid parameters. More time points should be set in future studies to decipher whether the current results are consistent in different metabolic states.

Another limitation needs to be considered when translating our findings into human studies. The equivalent dose of 200 mg/kg/day in mice for a 60 kg human is 972 mg/day based on body surface area conversion factors [48], which is not reasonably achievably through typical consumption of citrus peel which is used as a flavoring agent. The effects of low dose of NOB need to be verified in further animal or human studies, and the feasibility of NOB as a dietary supplement at this dose warrants more research.

## 5. Conclusions

NOB inhibited hepatic DNL and decreased liver TG in HFD-fed mice independently of liver *Bmal1*, whereas liver-specific *Bmal1* depletion reversed the beneficial effects of NOB on liver cholesterol homeostasis. The complex interactions between NOB, the circadian clock and lipid metabolism in the liver require further research.

## Data Availability

The data are available from the corresponding author upon reasonable request.

## Supplementary Materials

The following supporting information can be downloaded at: https://www.mdpi.com/article/10.3390/nu15112547/s1, Figure S1: PCR gel electrophoresis and WB was used to detect the expression level of BMAL1 in each organ of mice; Figure S2: Effects of liver-specific *Bmal1* knockout of body weight and liver weight parameters in NC-fed mice; Figure S3: Effects of liver-specific *Bmal1* knockout on the expression of clock and metabolic output genes in mice liver; Figure S4: Effect of NOB on the expression levels of clock and lipid metabolism genes in the liver of HFD-fed *Bmal1^flox/flox^* and *Bmal1*LKO mice; Table S1: Antibodies information; Table S2: The primers for the identification of knockout mice; Table S3: High-fat diet formulations; Table S4: Sequences of qRT-PCR primers used in relation to Figure 2, Figure 6, Figures S3 and S4.
