# Supplementary material for: Liver-Specific Bmal1 Depletion Reverses the Beneficial Effects of Nobiletin on Liver Cholesterol Homeostasis in Mice Fed with High-Fat Diet
Source: Nutrients. 2023 May 30;15(11):2547. doi: 10.3390/nu15112547 (PMC10255191; doi:10.3390/nu15112547)
Supplement: Supplementary file 1 [file nutrients-15-02547-s001.zip › nutrients-2403694-supplementary.pdf]

Supplementary Materials

Materials and Methods

Mouse Primary hepatocyte isolation

Mouse primary hepatocytes were isolated from *Bmal1*$^{flox/flox}$ or *Bmal1*LKO mice by perfusing liver with collagenase II (Gibco) at ZT12. Hepatocytes were resuspended in Dulbecco's Modified Eagle's Medium (DMEM, Gibco, Carlsbad, CA, USA) containing 10% (v/v) fetal bovine serum (Gibco, Carlsbad, CA, USA) and 1% (v/v) antibiotic (10,000 U/mL penicillin and 10,000 U/mL streptomycin, PanEra, Guangzhou, CHN), in a 5% CO_2 humidified environment at 37 °C and plated at a density of $1.5x10^6$ cells/35-mm dish.

Western blotting

The protein from liver or primary hepatocytes were lysis by RIPA lysis buffer and quantified by BCA protein quantitation kit (keygenbio). Approximately 40 μg isolated protein was separated electrophoretic ally by SDS-PAGE, and then transferred onto the polyvinylidene difluoride membranes (0.45 μm, Millipore, Bedford, MA, USA). After being blocked with 5% nonfat milk, membranes were successively incubated with specific primary antibodies and corresponding horseradish peroxidase-conjugated secondary antibodies (Table.S1). β-actin served as the internal reference. Quantification of protein bands was performed by Image J software (Bethesda, MD, USA).

Table S1. Antibodies information

Antibodies	Dilution	Corporation
BMAL1-specific primary antibody	1:1000	Cell Signaling Technology
β-actin-specific primary antibody	1:8000	Rayantibody
HRP Goat Anti-Mouse IgG (H+L)	1:8000	Rayantibody
HRP Goat Anti-Rabbit IgG (H+L)	1:8000	Rayantibody

Supplementary table

Table S2. The primers for the identification of knockout mice

Gene	Primer
Bmal1 loxp F5'-3'	TTGACTGTCTGTCAGTGCTTTCAT
Bmal1 loxp R5'-3'	TACCATGTTTATGGAGACTCTCAGC
Alb cre F5'-3'	GAAGCAGAAGCTTAGGAAGATGG
Alb cre R5'-3'	TTGGCCCCTTACCATAACTG

Table S3. High-fat diet formulations

Class description	Ingredients	content
Protein	Casein, Lactic, 30 Mesh	200.00 g

Protein	Cystine, L	3.00 g
Carbohydrate	Sucrose, Fine Granulated	68.80 g
Carbohydrate	Starch, Corn	
Carbohydrate	Maltodextrin 10	125.00 g
Fiber	Solka Floc, FCC200	50.00 g
Fat	Soybean Oil, USP	25.00 g
Fat	Lard	245.00 g
Mineral	S10026B	50.00 g
Vitamin	Choline Bitartrate	2.00 g
Vitamin	V10001C	1.00 g
Dye	Dye, Yellow FD&C #5, Alum. Lake 35-42%	0.05 g
	Total:	773.85 g
Caloric Information Physiological Fuel Values:		
	Protein	20 % Kcal
	Fat	60 % Kcal
	Carbohydrate	20 % Kcal
	Energy density	5.21 Kcal/g

Table S4. Related to Figures 2 and 6. Sequences of qPCR primers used.

Gene Name	Forward Primer	Reverse Primer
β-actin	GGCTGTATTCCCCTCCATCG	GGCTGTATTCCCCTCCATCG
mBmal1	ACAGTCAGATTGAAAAGAGGCG	ACAGTCAGATTGAAAAGAGGCG
mClock	TTTTCCAGGGCACAAGTC	CATTCCCAGCAGCACAT
mRev-erbα	CTCTCTGCTCTTCCCATGC	CTTGGGGTGGCTATACTGC
mRev-erbβ	TGAACGCAGGAGGTGTGATTG	GAGGACTGGAAGCTATTCTCAGA
mCry1	ACAAACAACCCACGCTTT	GTCAGGAAACAGGCAACC
mCry2	GCGTCTGTTTGTAGTCCGGG	TCCCAAAGGGTTCAGAGTCATA
mPer1	CACCAAGCTGCCTCTTCC	CATGAGGGGTCGGTCCT
mPer2	CAGGTTGAGGGCATTACCTCC	AGGCGTCCTTCTTACAGTGAA
mRorα	GTGGAGACAAATCGTCAGGAAT	TGGTCCGATCAATCAAACAGTTC
mRorγ	CGCGGAGCAGACACACTTA	CCCTGGACCTCTGTTTTGGC
mSrebp1c	CAAGGCCATCGACTACATCCG	CACCACTTCGGGTTTCATGC
mPparγ	GGAAGACCACTCGCATTCCTT	GGAAGACCACTCGCATTCCTT
mPparα	CATTTCTCCTTGGCGTGT	CCTCAGACCTTGCTTTGG
mPpargc1α	TATGGAGTGACATAGAGTGTGCT	GTCGCTACACCACTTCAATCC
mAcaca	AATGAACGTGCAATCCGATTTG	AATGAACGTGCAATCCGATTTG
mFasn	GGAGGTGGTGATAGCCGGTAT	TGGGTAATCCATAGAGCCCAG
mScd1	TTCTTGCGATACACTCTGGTGC	TTCTTGCGATACACTCTGGTGC
mDgat2	GCGCTACTTCCGAGACTACTT	GGGCCTTATGCCAGGAAACT
mCpt1a	TGGCATCATCACTGGTGTGTT	GTCTAGGGTCCGATTGATCTTTG
mHmgcr	TGTTCACCGGCAACAACAAGA	CCGCGTTATCGTCAGGATGA
mCyp7a1	GAACCTCCTTTGGACAACGGG	GGAGTTTGTGATGAAGTGGACAT
mCyp7b1	GGAGCCACGACCCTAGATG	GCCATGCCAAGATAAGGAAGC
mCyp8b1	CACGGGGATGTCTTCACGG	TGAGCACCAGTTCTTTTGCATAG

Gene Name	Forward Primer	Reverse Primer
mAcox1	CCGCCACCTTCAATCCAGAG	CAAGTTCTCGATTTCTCGACGG
mGata4	CACCCCAATCTCGATATGTTTGA	GCACAGGTAGTGTCCCGTC
mAbcg5	AGAGGGCCTCACATCAACAGA	CTGACGCTGTAGGACACATGC
mShp	CAGGTCGTCCGACTATTCTGT	AGGCTACTGTCTTGGCTAGGA
mMttp	AATGCGGGTCAACAGAGAGG	CTGGCTCGTTTTCATAGGAGTAG
mDgat1	CTGATCCTGAGTAATGCAAGGTT	TGGATGCAATAATCACGCATGG

Supplementary figure

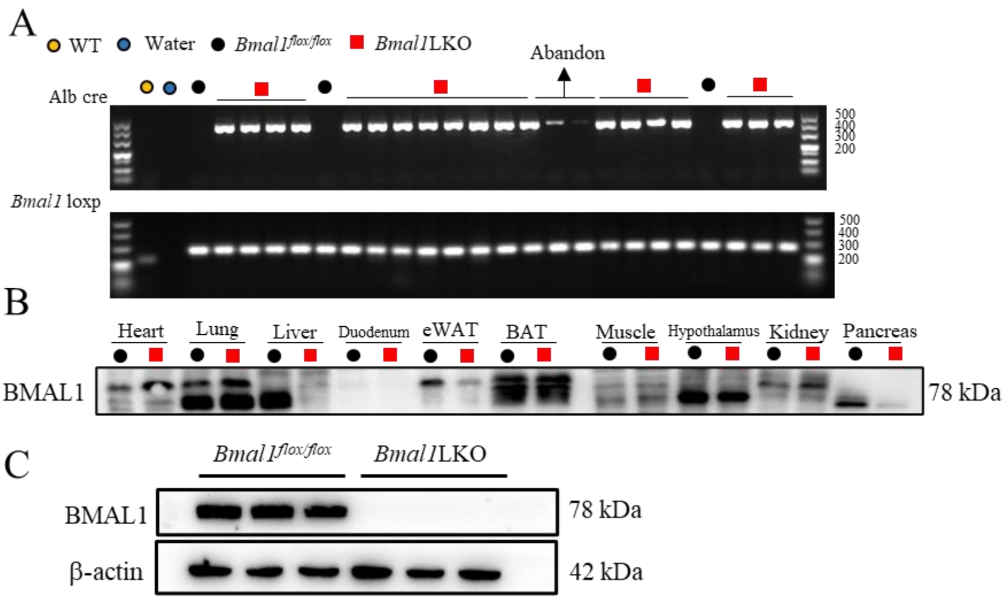

Figure S1. PCR gel electrophoresis was used to identification mouse genotype (A). Western blot was used to detect the expression level of BMAL1 in each organ of mice. The expression level of BMAL1 in heart, lung, liver, duodenum, epididymis white adipose tissue (eWAT), brown adipose tissue (BAT), musle, hypothalamus, kidney, pancreas was detected by western blot (B). The expression level of BMAL1 in primary hepatocytes (C).

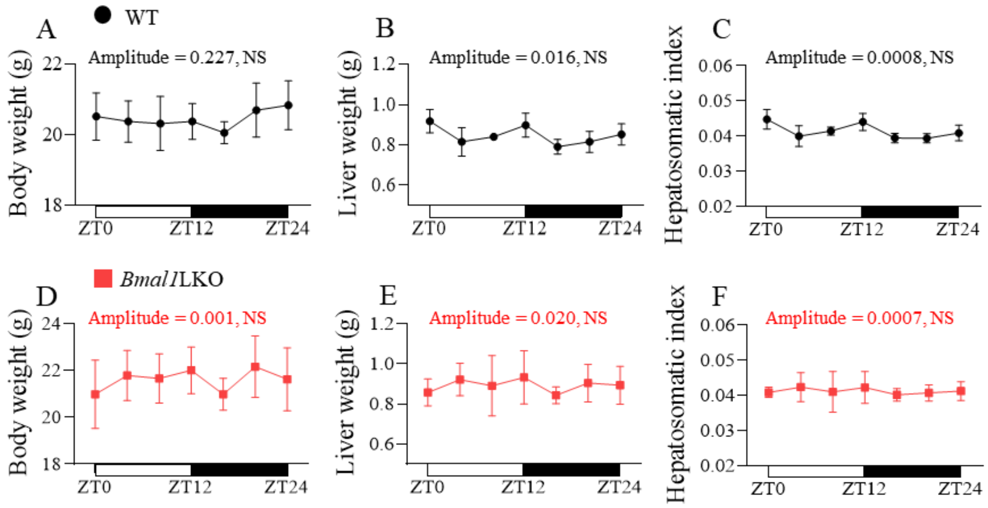

Figure S2. Related to Figure 1. Effects of liver-specific *Bmal1* knockout of body weight and liver

weight parameters in NC-fed mice**.** Body weight (A and D), liver weight (B and E) and liver/body weight (C and F) in NC-fed WT and *Bmal1*LKO mice at seven time points. Data are presented as mean ± SD (n = 5 per group at each time point). NS indicates that the 24-h rhythmicity is not significant.

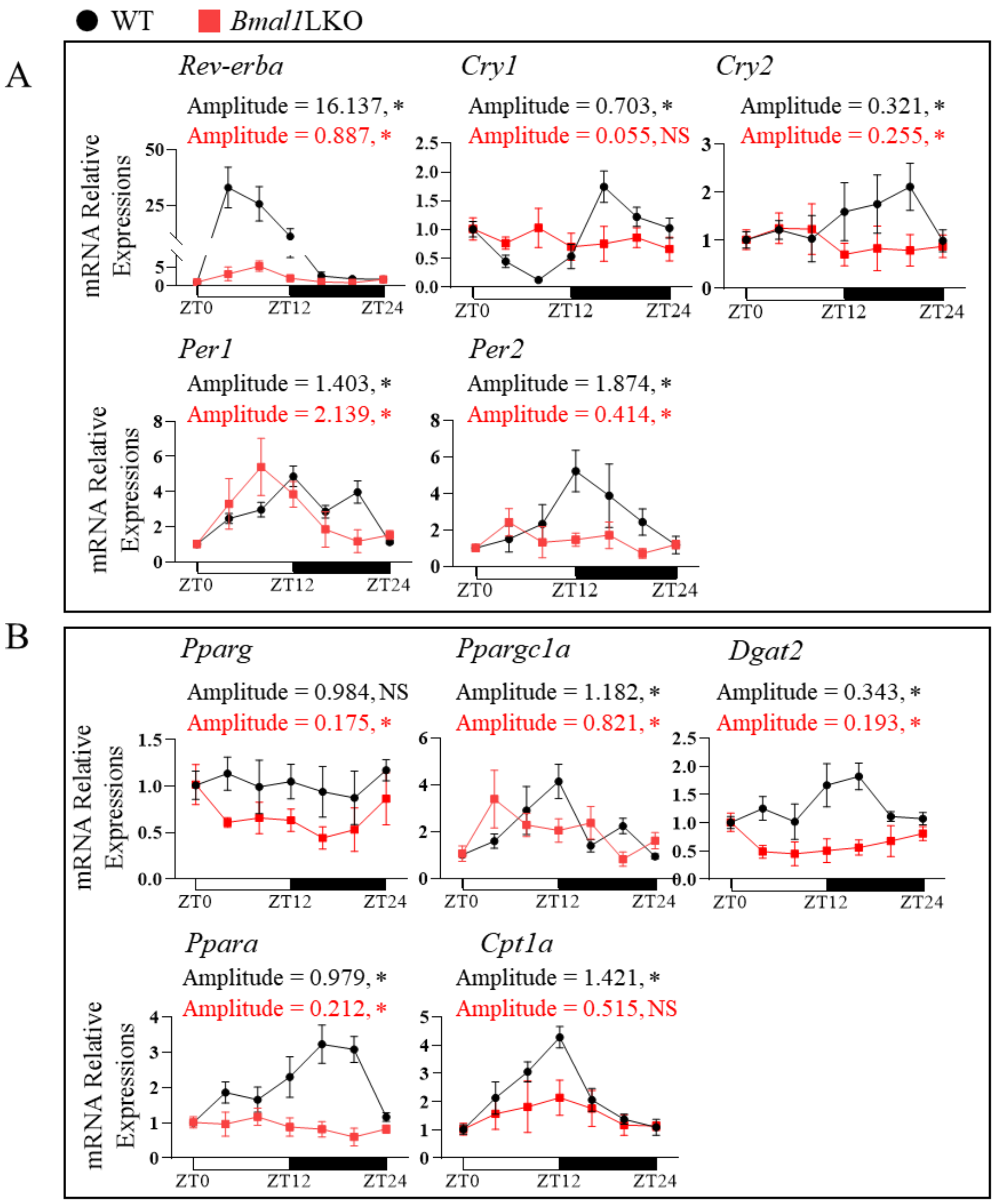

Figure S3. Related to Figure 2. Effects of liver-specific *Bmal1* knockout on the expression of clock and metabolic output genes in mice liver. qRT-PCR analyses of mRNA expression of clock genes (A), lipid metabolism genes (B), in the liver of NC-fed WT and *Bmal1*LKO mice at seven time points. Data are presented as mean ± SD (n = 5 per group at each time point). ∗*P* < 0.05 indicates that the 24-h rhythmicity is significant.

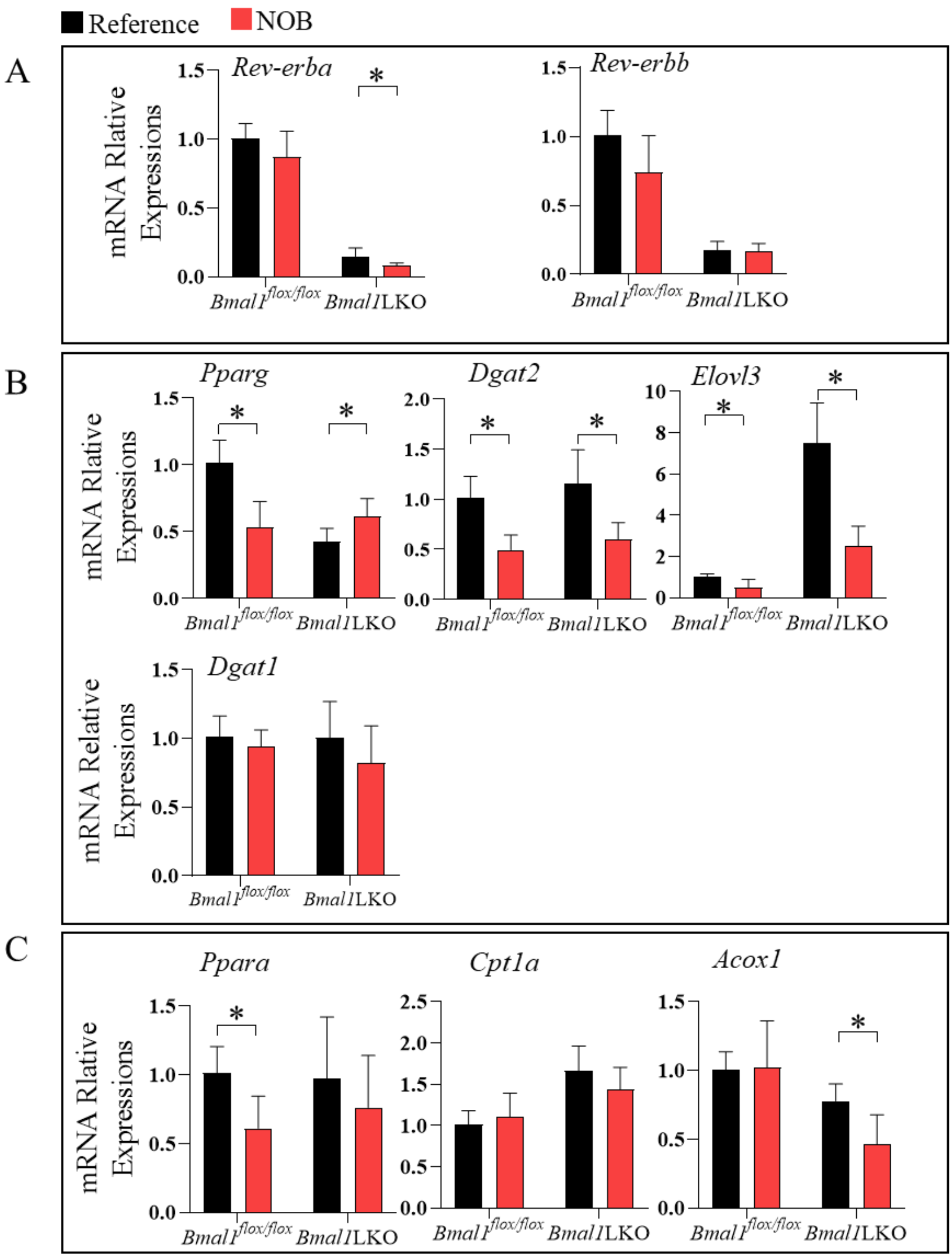

Figure S4. Related to Figure 6. Effect of NOB on the expression levels of clock and lipid metabolism genes in the liver of HFD-fed *Bmal1*$^{flox/flox}$ and *Bmal1*LKO mice. qRT-PCR analyses of mRNA expression of clock genes (A), lipid synthesis genes (B) and lipid oxidation genes (C) in the liver of HFD-fed *Bmal1*$^{flox/flox}$ and *Bmal1*LKO mice with reference or NOB treatment. Data are presented as mean ± SD (n = 5 per group). $*P < 0.05$, *flox/flox*.reference versus *flox/flox*.NOB or LKO.reference versus LKO.NOB.
